# Modelling ‘Type B’ ejecta formation reveals reactor Unit 1 conditions during the Fukushima Daiichi Nuclear Disaster

**DOI:** 10.1038/s41598-023-30903-6

**Published:** 2023-03-06

**Authors:** Lior A. S. Carno, Jack J. Turner, Peter G. Martin

**Affiliations:** 1 HH Wills Physics Laboratory, School of Physics, University of Bristol, Tyndall Avenue, Bristol, BS8 1TL UK; 2 HH Wills Physics Laboratory, Interface Analysis Centre, School of Physics, University of Bristol, Tyndall Avenue, Bristol, BS8 1TL UK

**Keywords:** Environmental sciences, Pollution remediation, Geochemistry, Volcanology, Nuclear energy, Glasses, Mechanical properties, Fluid dynamics

## Abstract

For the first time, a model was developed to simulate the cooling of the Fukushima Daiichi Nuclear Power Plant reactor Unit 1-derived, ‘Type B’ radiocaesium bearing microparticles, distributed into the environment during the 2011 nuclear meltdown. By establishing an analogy between ‘Type B’ CsMP and volcanic pyroclasts, the presented model simulates the rapid cooling of an effervescent silicate melt fragment upon atmospheric release. The model successfully reproduced the bi-modal distribution of internal void diameters observed in ‘Type B’ CsMP, however, discrepancies resulted primarily due to the neglection of surface tension and internal void coalescence. The model was subsequently utilised to estimate the temperature within reactor Unit 1 in the instant preceding the hydrogen explosion—between 1900 and 1980 K. Such a model demonstrates the accuracy of the volcanic pyroclast—‘Type B’ CsMP analogue, and confirms radial variations in cooling rate as the cause of the vesicular texture of Unit 1 ejecta. The presented findings provide scope to further explore the comparison between volcanic pyroclasts and ‘Type B’ CsMP via experimentation, which will provide a deeper understanding of the specific conditions within reactor Unit 1 during the catastrophic meltdown at the Japanese coastal plant.

## Introduction

### Fukushima Daiichi nuclear disaster

**Figure 1:**
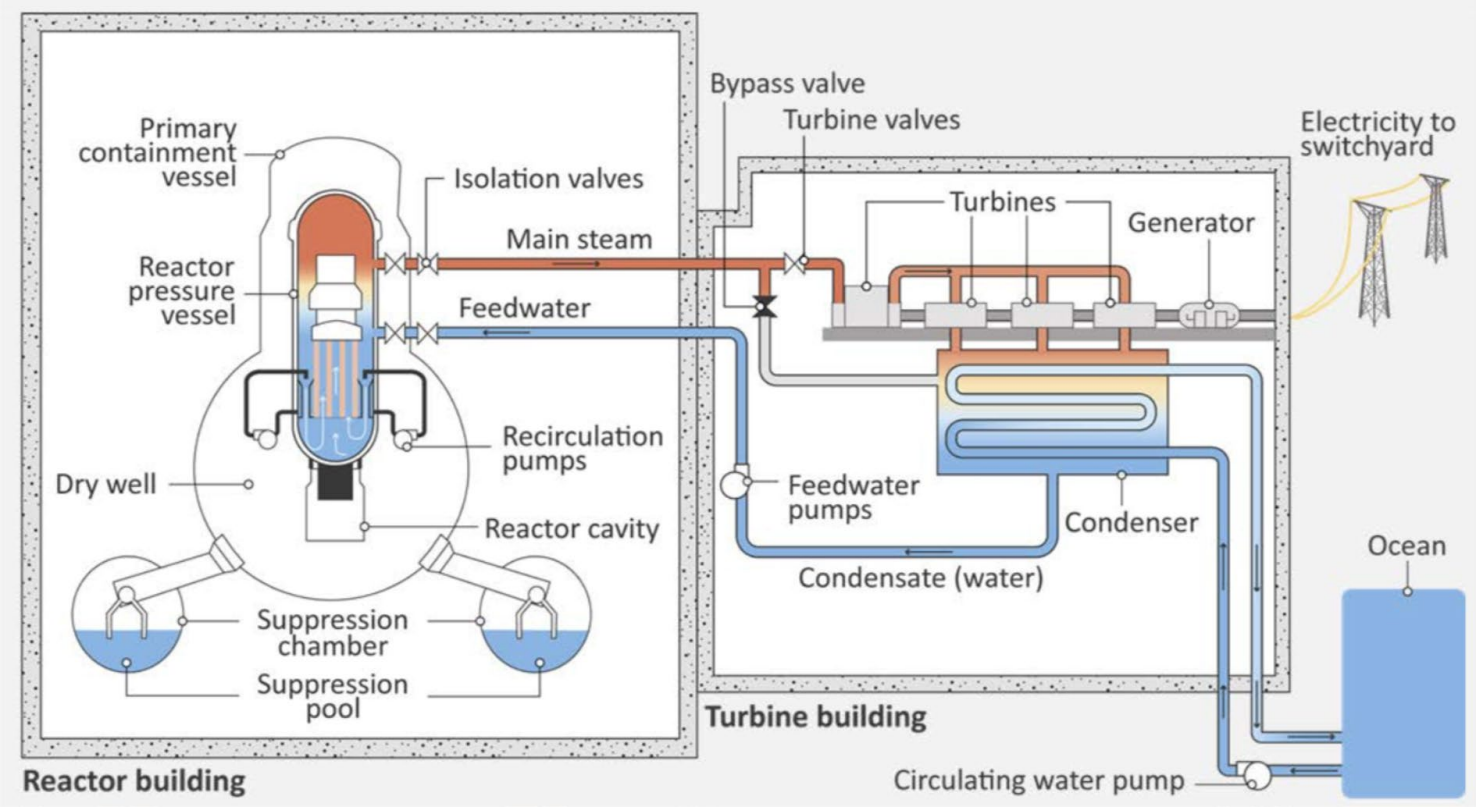
Schematic of the Mark-I containment associated with the boiling water reactors (BWR) used in FDNPP Units 1–5. Water is used both as coolant and as a neutron moderator (via its flow rate) alongside neutron absorbing control rods to control reactivity. Pure uranium oxide (UO_2_) was used as the nuclear fuel in reactor Units 1 and 2, while a component of mixed oxide (MOX) was used in reactor Unit 3^2^. These fuel elements are encased within zirconium cladding (Zircaloy-4), and the primary containment assembly and pipes for the heat exchanger network are clad with a Rockwool type insulation. Reproduced with permission from IAEA^4^.

On the 11th March 2011, the magnitude 9.0 Great Tōhoku Earthquake occurred off the eastern coast of Japan. The resultant tsunami inundated 560 km^2^ of land, destroying over a million buildings and killing approximately 19,000 people^1,2^. The economic damage was estimated at US$ 235 billion, making it the most costly environmental disaster in history^3^. Located 180 km from the earthquake’s epicentre, the Fukushima Daiichi Nuclear Power Plant (FDNPP) comprised six boiling water reactors, shown schematically in Figure 1, operated by Tokyo Electric Power Company (TEPCO). Upon detection of the earthquake at 14:46 Japan Standard Time (JST)^4^, all three operational reactors at FDNPP, Units 1, 2 and 3 (Units 4, 5, 6 were offline at the time), immediately shut down via insertion of fission-inhibiting control rods (also known as safety control rod axe man - ‘SCRAM’). While the station proved robust seismically, the earthquake damaged off-site power transmission infrastructure, forcing the plant to switch to emergency diesel generators. These failed 40 mins later, when the entire site was inundated by a 15 m high tsunami wave, resulting in station-wide power loss^5^. Additionally, the seawater pumps, residual heat removal systems and electrical switch gear were all destroyed by the tsunami, disabling all of the plants core-cooling capabilities. One hour after ‘SCRAM’, the three operational reactors were still producing approximately 1.5% of their nominal thermal output via fission product decay^2^. Isolated from their ultimate heat-sink, the temperature and pressure within the reactor pressure vessels (RPV) rapidly increased, yielding large quantities of steam. In addition, the exothermic interaction of zirconium cladding with this super-heated steam produced an estimated 130 kg of hydrogen in reactor Unit 1^6^. Various attempts to alleviate the growing pressure and cool each of the reactor cores progressively failed, culminating in core meltdowns. On 12th and 14th March, hydrogen explosions occurred in FDNPP Units 1 and 3, respectively, blowing the roofs from both reactor buildings. Reactor Unit 4, despite not being in operation, also exploded due to an influx of the combustible gas being vented from the nearby reactor Unit 3^7^. Although the Unit 2 reactor building did not explode, on 15th March its primary containment vessel (PCV) developed a leak^2^, releasing the incident’s largest contribution of on-land radioactive contamination^8^.

### Radioactive release

**Figure 2:**
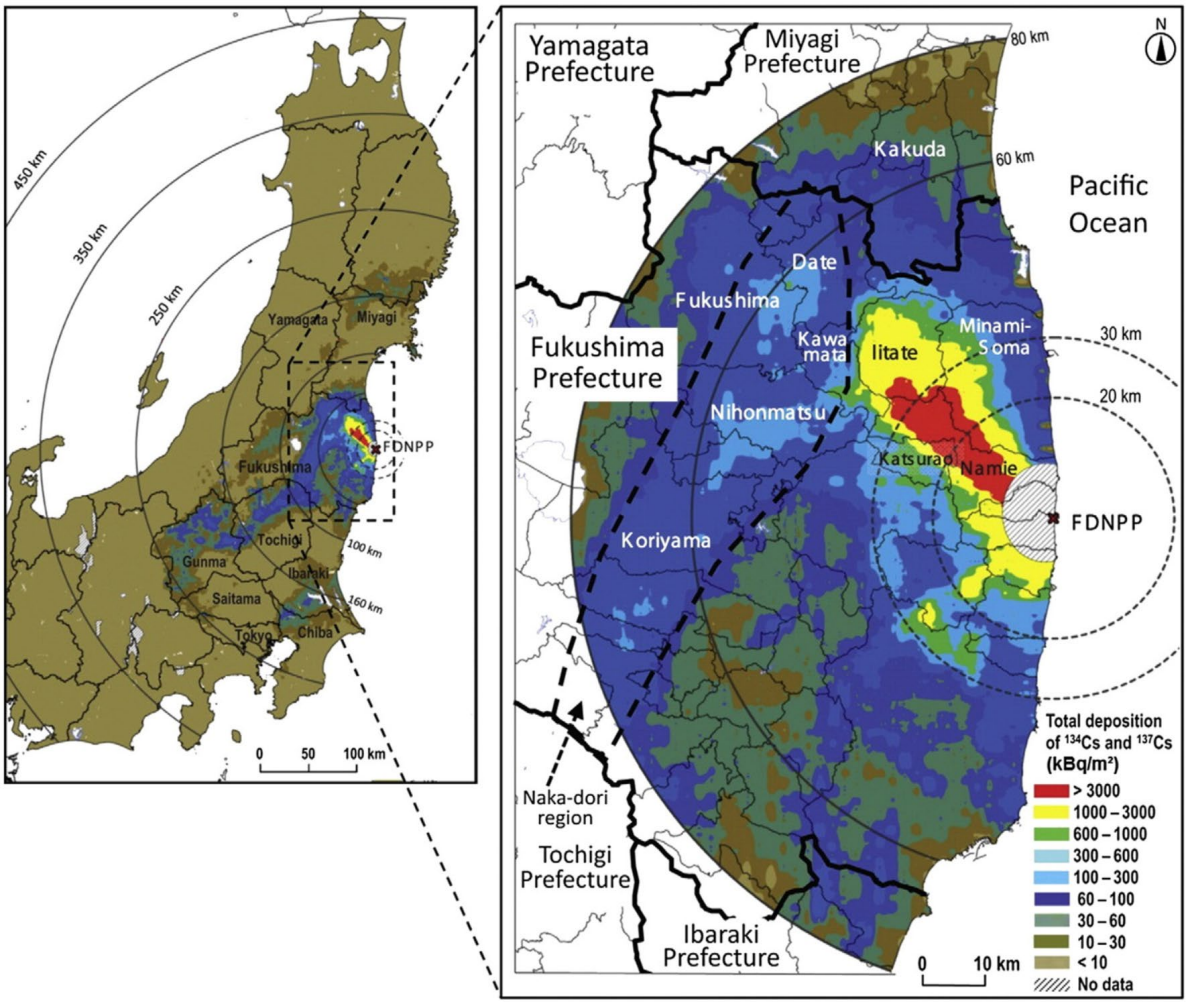
Estimated total distribution of radiocaesium after the FDNPP accident. The narrow red band corresponds to the highest activity region of the primary containment plume that was released north west from Unit 2 of the FDNPP^9^. Adapted from^20^.

The FDNPP accident, rated 7, the highest level, on the International Nuclear and Radiological Event Scale (INES), released an estimated total of 340–800 PBq of radioactivity to the surrounding environment. This was approximately one tenth of the radiation released during the 1986 Chernobyl Nuclear Power Plant disaster^9^. Cumulative releases from the three FDNPP reactor units, combined with their intermittent cooling histories, resulted in a diverse suite of emissions of far greater complexity than the single release episode during the Chernobyl disaster^8^. The released radionuclides spread throughout Japan, as seen in Figure 2, and consisted mainly of the radiocaesium isotopes, $^{134}$Cs and $^{137}$Cs, with half-lives of 2.06 and 30.07 years, respectively, and radioiodine, $^{131}$I, with a considerably shorter half-life of 8.02 days^10^. Due to its 8 day half-life, the latter quickly decayed out of the environment, leaving radiocaesium as the primary gamma-emitting pollutant.

Following the accident, a soluble form of radiocaesium was widely detected in the surrounding soils, rivers and plants^11^. The insoluble form of Fukushima-derived radiocaesium was first identified in the environment two years after the accident by Adachi et al.^12^. Termed ‘caesium-bearing microparticles’ (CsMP), these micron-scale particles are SiO$_{2}$-based with a high specific radioactivity. Their glassy-state make them resistant to erosion processes, meaning they present a far greater sustained radiation hazard than the soluble form of radiocaesium^13,14^. Because CsMP were formed inside the reactors during the FDNPP accident, their properties provide critical insight into the meltdown chronology and conditions^15^. The study of CsMP is therefore vital for assessing the extent of reactor damage and planning for their decommissioning, as well as to the cleanup of the contaminated areas surrounding FDNPP.

CsMP have been broadly classified into two groups: ‘Type A’ and ‘Type B’, originating from reactor Unit 2 and Unit 1, respectively^16,17^. These groups are characterised primarily by their varying $^{134}$Cs/$^{137}$Cs activity ratio, which arises due to the differing fuel burn-up from each reactor. Via comparison of these measured $^{134}$Cs/$^{137}$Cs ratios with those calculated analytically using reactor-core inventory modelling^18^, the source reactors of ‘Type A’ and ‘Type B’ CsMP have been successfully identified. In addition to their Cs activity ratio, the two CsMP types are distinguishable by their morphology. ‘Type A’ particles are smaller, ranging in size from 2 to 10 μm and highly spherical, thus are commonly termed ‘Cs-balls’^2^. In contrast, ‘Type B’ material is larger, with particle diameters ranging from 50 to 400 μm^16^, and generally more angular, although spherical suites of ‘Type B’ particles have been identified^19^. Moreover, different CsMP are prevalent in certain locations; ‘Type A’ particles in the western regions and ‘Type B’ particles in the northern areas close to the FDNPP site^16^. A summary of the differences between ‘Type A’ and ‘Type B’ particles is given in Table 1. Due to their narrow spatial distribution and proximity to the FDNPP site (≤ 8 km), obtaining large numbers of such ‘Type B’ particles has proved challenging. As a result, there exists limited research regarding ‘Type B’ material in contrast with the comprehensively studied ‘Type A’ particles (see e.g.^11^). For this reason, the chosen focus of this research is the formation of ‘Type B’ CsMP, rather than the more abundant ‘Type A’ material.

**Table 1:** Comparison of ‘Type A’ and ‘Type B’ particle properties. Adapted from^16,19^.

Characteristic	‘Type A’	‘Type B’
Size distribution (observed)	1–10 μm	70–400 μm
Cs activity ratio (mean)	1.04	0.93
Source reactor(s)	Unit 2/3	Unit 1
Emission date (estimate)	March 15th, 2011	March 12th, 2011
$^{137}$ Cs activity (Bq/particle)	$\sim$ 10$^{-2}$−10$^2$	$\sim$ 10$^{1}$−10$^4$
Geographical distribution	Wide	Limited

**Figure 3:**
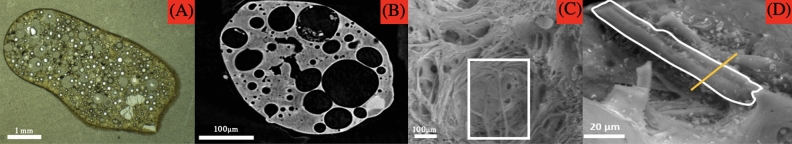
(**A**) Photomicrograph of a ‘Pele’s tear,’ cut along the direction of elongation, showing highly spherical, internal vesicles which decrease in size towards the particle’s edge, from^23^. (**B**) Orthogonal X-ray tomographic (absorption contrast) section of a ‘Type B’ CsMP displaying the presence of many, different sized voids within the particle, comparable in its internal structure to (**A**), from^21^. (**C**) Scanning electron microscope (SEM) image of the internal structure of volcanic pumice, revealing fibrous features (marked by the white square), from^24^. (**D**) SEM image of the surface of ‘Type B’ CsMP, marked are the fibrous features, akin to the fibrous features observed in (**C**), from^22^.

**Figure 4:**
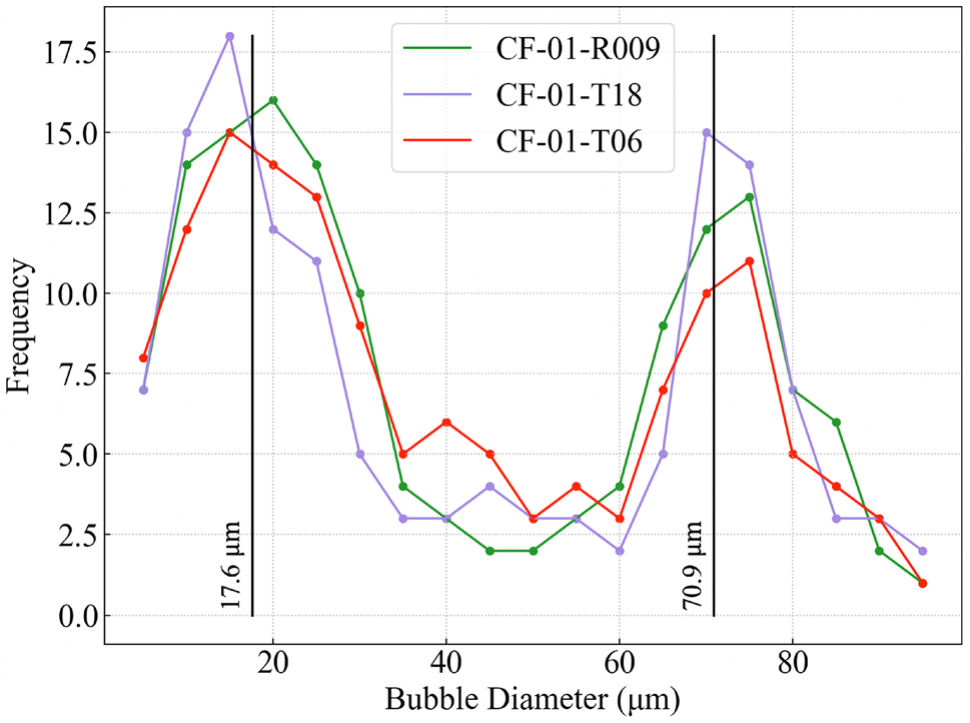
Plot of void diameter against frequency demonstrating the bi-modal void size distribution within ‘Type B’ CsMP. The first peak, located at 17.6 μm, represents the incorporated fission product bubbles, ‘frozen’ around the particle circumference due to quench-like cooling. The second peak, centred at 70.9 μm, shows the increased diameter of the gaseous voids as a result of depressurisation and coalescence. Adapted from^21^.

### ‘Type B’ ejecta material

Energy Dispersive Spectroscopy (EDS) analysis has identified Si (primarily as silicate) as the main constituent of ‘Type B’ CsMP (24.9–37.1 wt%), whilst synchrotron characterization has revealed a highly heterogeneous distribution of other elemental constituents (including Mo, Fe, Ni, Cd, Sn, and Cr)^16,21,22^. It has been proposed that unlike ‘Type A’ CsMP, which are most likely derived from SiO condensate produced in molten core-concrete interactions, these larger CsMP were formed from the melting and amalgamation of fibrous (Si-based) Rockwool thermal insulation surrounding the RPV^16^. Artefacts of this fibrous material are observed as inclusions on the surface of ‘Type B’ CsMP, as highlighted in Figure 3D. These exhibit a uniform orientation across the particle surface, suggesting the CsMP are the result of a violent emission event such as the hydrogen explosion that occurred on 12th March 2011^22^.

Initial examination using scanning electron microscopy (SEM) showed the surface of ‘Type B’ CsMP consist of smooth sections interrupted by numerous micron-scale spherical voids^16,22^. More detailed investigations into the internal 3D structure via SR-μ-XRF and X-ray tomography (XRT) identified a significant internal volume (24–31%) of spherical voids which display a bi-modal diameter distribution, as shown in Fig. 4.^19,21,25^. The smaller voids, with a mean diameter = 17.6 μm, are concentrated around the circumference of CsMP. These are believed to be caused by the incorporation of the prevalent gas within the reactor, composed of fission products (Cs, Sb, Rb), noble gases and hydrogen, into the molten silicate due to the considerable overpressure of this gas. In contrast, the larger, more centrally located voids, with a mean diameter = 70.9 μm, likely originate from the release of trapped volatile species from silicate fibres into the bulk particle^21^. CsMP with higher porosities were in fact found to have higher $^{137}$Cs radioactivity, indicating they captured larger amounts of these volatile fission products during their formation^19^. The bi-modal distribution of void diameters is likely explained by the radially varying cooling rate within the CsMP. Following the hydrogen explosion of reactor Unit 1, molten CsMP were ejected from the PCV, where they formed, into the environment. Upon exposure to the atmosphere, the CsMP surfaces were cooled almost instantly. This caused the exterior silicate melt to solidify, creating a porous outer rind with voids ‘frozen’ in place. The internal particle bulk cooled at a somewhat slower rate, allowing for void coalescence as well as significant expansion of the more centrally located voids owing to depressurisation^21^.

### Volcanic ejecta analogy

To date, no studies have been conducted which investigate the formation mechanisms of ‘Type B’ CsMP. However, the formation of volcanic ejecta material, specifically pyroclastic bombs, is well documented and multiple studies have successfully used the thermal history of these ejecta to examine eruption dynamics^26–28^. Such volcanic bombs are found as ballistically emplaced clasts in the crater or on the flank of volcanoes or in pyrcoclastic flow deposits kilometers away from the vents (Guagua Pichincha^27^, Montserrat^28^, Tungurahua^29,30^). Despite being several orders of magnitude larger than ‘Type B’ CsMP, volcanic bombs share many key properties with the Unit 1 particulate released in the Fukushima disaster. Like ‘Type B’ CsMP, this form of pyroclast starts as part of an effervescent mass of silicate melt within the magmatic shaft of a volcano. During explosive eruptions, this bulk is ejected into the atmosphere as small fragments of melt that experience rapid cooling and depressurisation^31^. During this phase, volatile exsolution and bubble growth commence, but are quickly slowed by the increase in magmatic viscosity of the melt. The glass transition temperature, $T_g$, is the kinetic limit at which a material transitions from a viscous liquid to a glass^32^. Different depths within the clast cross $T_g$ at different times, resulting in a radial bubble size distribution that provides a textural record of the clast’s thermal history. The final morphology of solidified ejecta from both FDNPP Unit 1 and volcanic eruptions are closely related. Both are composed primarily of silicate; $\sim$ 64–69 wt% in ‘Type B’ CsMP and $\sim$ 56–65 wt% in volcanic bombs^30,33^. Moreover, the vesicular interior surrounded by a dense rind of small bubbles observed in the pyroclasts is also very similar to the morphology of ‘Type B’ CsMP, as demonstrated in Fig. 3.

We therefore hypothesise that ‘Type B’ CsMP experience identical formation mechanisms to volcanic bombs^26,34,35^. The implementation of this volcanic bomb analogy enables the utilisation of volcanology research and the adaptation of volcanic bomb cooling models to construct a simulation of ‘Type B’ CsMP cooling. This is subsequently used to further investigate CsMP material properties and hence aid their removal from the environment, as well as estimate the conditions within reactor Unit 1 during the FDNPP accident.

## Methods

### Model compilation

**Figure 5:**
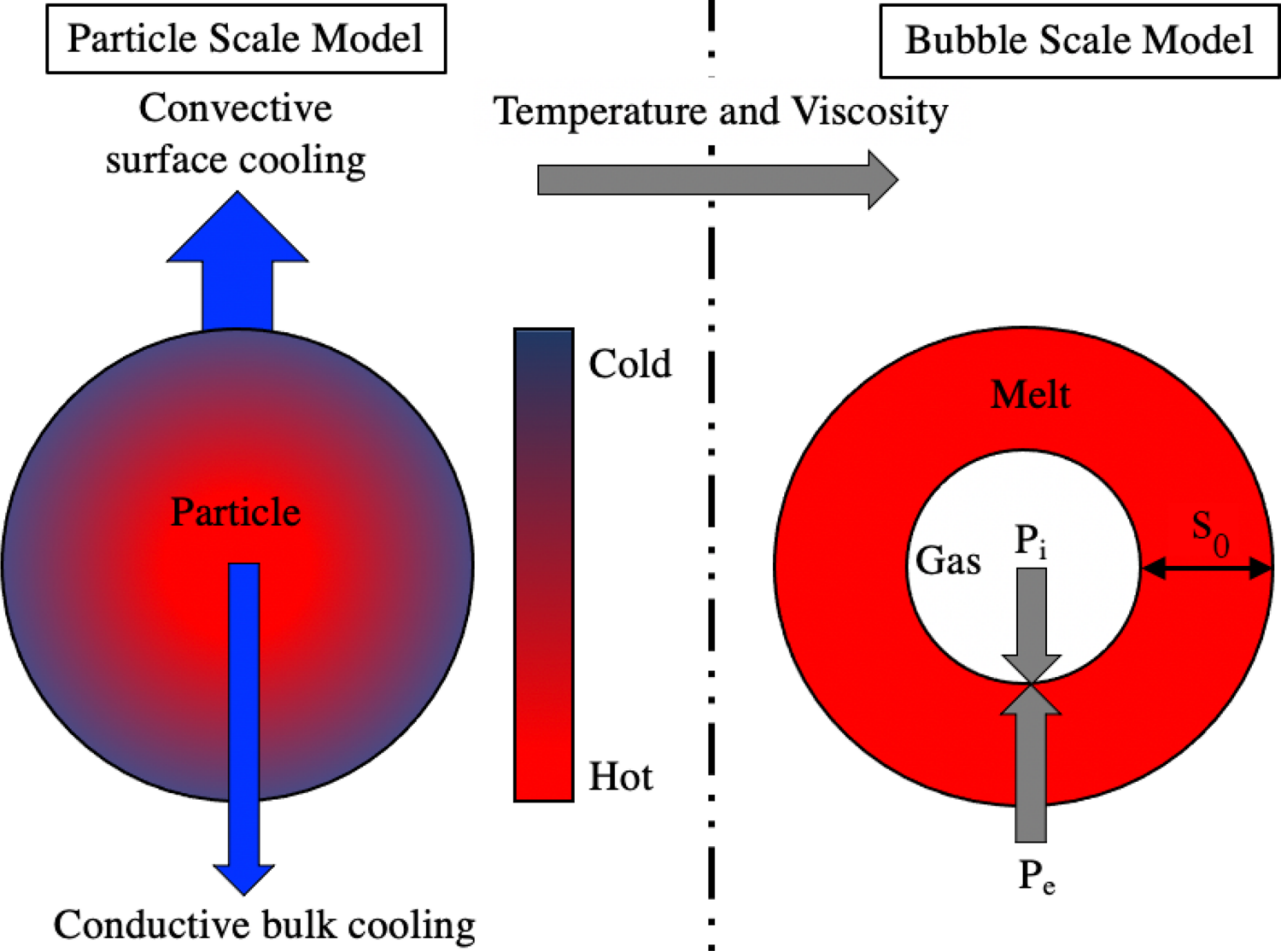
Schematic outlining the physical processes at each scale of the model. Particle-scale: the cooling of the isotropic, spherical particle from convective heat transfer to the surrounding environment and conductive cooling within the particle. Bubble-scale: The bubble growth model in which growth is limited by viscosity and halted once the temperature is lower then the glass transition temperature.

The constructed model simulates a ‘Type B’ CsMP cooling upon atmospheric release due to the Unit 1 hydrogen explosion. The model operates on two scales; the particle-scale model captures radial temperature and viscosity changes of the CsMP while the bubble-scale model computes internal bubble growth. These two scales were then coupled through the melt viscosity, which limits bubble growth, to obtain a complete model of ‘Type B’ CsMP formation. At both scales it is assumed that melt fragments are spherical, isotropic and of uniform composition. A model schematic is shown in Fig. 5.

#### Particle cooling

Upon exposure to the atmosphere following the hydrogen explosion, CsMP experienced rapid cooling. It was assumed convective heat loss occurred only at the surface, with conductive cooling due to the heat gradient formed between the centre and surface^36^ dominating within the particle bulk. To further simplify the model, radiative heat loss at the particle surface was taken to be negligible. The radial and temporal heat profile of the particle due to just conductive heat loss was then modelled by solving the one-dimensional, spherically symmetric heat equation

1 $$\begin{aligned} \rho _{p} c_{p} \frac{\partial T}{\partial t} = \frac{k_{p}}{r^2} \frac{\partial }{\partial r} \left( r^2 \frac{\partial T}{\partial r}\right) , \end{aligned}$$

where $\rho _{p}$ is density of the particle, $c_{p}$ the heat capacity of the particle, $k_{p}$ the thermal conductivity and *r* the radial coordinate. The physical particle properties (e.g. density, heat capacity, thermal conductivity) were assumed to be constant throughout the model. The convective heat loss at the particle surface was accounted for by imposing the boundary condition

2 $$\begin{aligned} k_{p}\left( \frac{ \partial T}{\partial r}\right) _{r=R} = -q_c, \end{aligned}$$

where

3 $$\begin{aligned} q_c = h( T_\infty - T_s) \end{aligned}$$

is the convective heat flux. *h* is the heat transfer coefficient, $T_s$ is the surface temperature of the particle and $T_ \infty$ the temperature of the surroundings^23^. The heat transfer coefficient was calculated using the equation

4 $$\begin{aligned} h = \frac{{\text {Nu}} k_g}{2 r_p}, \end{aligned}$$

where Nu is the Nusselt number, $k_g$ the thermal conductivity of the air surrounding the particle and $r_p$ the particle radius. Calculation of the Nusselt number required the particle Reynolds number, Re, determined by

5 $$\begin{aligned} {\text {Re}} = \frac{2 |(v_g - v_p)| r_p \rho _g}{\eta _g}, \end{aligned}$$

where $v_g$ is the velocity of the surrounding air, $v_p$ the particle velocity, $\rho _g$ the air density and $\eta _g$ the air viscosity. In previous studies^26^, the Nusselt number was calculated for a negligible internal temperature gradient by invoking the lumped capacitance approximation. This states that for Biot numbers close to zero (Bi$\rightarrow 0$) , the convective heat transfer to the surrounding gas limits the surface heat flux, and internal conduction is large enough to equilibrate the internal temperature gradient of the pyroclast. This contradicts our key hypothesis that radial variations in viscosity caused the unique internal texture observed in ‘Type B’ CsMP. For this reason the Nusselt number used in this study was calculated using newer data from Moitra et al.^37^, that is not dependent on the lumped capacitance approximation:

6 $$\begin{aligned} \textrm{Nu}=a+b {\text {Re}}^{1 / 2} {\text {Pr}}^{1 / 3} \end{aligned}$$

with fitting parameters $a=76$ and $b=1.9$, and Pr is the Prandtl number, taken to be 0.71 for ambient air^37^.

#### Particle viscosity

The viscosity of molten silicate is the dominant control on bubble growth rate within a melt fragment ejected via the hydrogen explosion. It is strongly dependent on the melt temperature and composition, and can vary over 15 orders of magnitude throughout particle cooling^38^. This change was modelled using the empirical Vogel–Fulcher–Tamman (VFT) model

7 $$\begin{aligned} log(\eta ) = A + \frac{B}{T - C}, \end{aligned}$$

where $\eta$ is viscosity, *T* is temperature and *A*, *B* and *C* are empirically determined constants. The parameter *A* represents the melt viscosity at an infinite temperature and can, to high degrees of accuracy, be considered independent of melt composition^38^. However, this is not the case for *B* and *C*, which are related to melt composition and calculated as follows:

8 $$\begin{aligned} B= & {} \sum _{n=1}^{7} [b_i M_i] + \sum _{j = 1}^{3} [b_{1j}(M1_{1j} \cdot M2_{1j})], \end{aligned}$$

9 $$\begin{aligned} C= & {} \sum _{n=1}^{6} [c_i N_i] + [c_{11}(N1_{11} \cdot N2_{11})] \end{aligned}$$

where *M* and *N* represent the weight percentage of a given chemical component and *b* and *c* are empirically determined optimization parameters^38^.

#### Bubble growth

The bubble growth rate decreased depending on the depth within the particle during cooling. Therefore, bubble size and position provide valuable insight into the cooling history of the CsMP. Since the bubbles were assumed to be perfectly spherical, radial bubble growth was modelled in one-dimension. By assuming the bubble growth was limited by the viscosity of a thin shell of surrounding melt only, radial growth was described using the equation

10 $$\begin{aligned} \frac{dR}{dt} = \frac{R^2}{12 \eta {s}} (P_i - P_e), \end{aligned}$$

where *R* is bubble radius, *t* time, $\eta$ the viscosity of the surrounding melt, *s* the shell thickness, $P_i$ the outwardly directed internal bubble pressure and $P_e$ the external pressure experienced by the bubble. Surface tension provides an additional pressure acting towards the bubble centre. However, this is only important during the early stages of bubble growth^39^, and so has been treated as negligible in the presented model. The full derivation for Eq. (10) can be found in Appendix A. By assuming the ideal gas law and isothermal gas conditions, initial internal bubble pressure, $P_0$, can be related to internal bubble pressure at some arbitrary time after depressurisation has occurred through the relation

11 $$\begin{aligned} P_i R^3 = P_0 R_0^3, \end{aligned}$$

where $R_0$ is initial bubble radius. By fixing the volume of the shell of surrounding melt, *s*, and assuming the initial shell of melt has thickness significantly less than the radius of the bubble which it surrounds ($s_0 \ll R_0$), bubble radius can be expressed as

12 $$\begin{aligned} R^2 = \frac{R _0^2 {s_0}}{r}, \end{aligned}$$

where $s_0$ is the initial thickness of the melt surrounding the pore and *r* is the radial co-ordinate. By combining Eqs. (10)–(12), a final expression which was used to model radial bubble growth was obtained^40^:

13 $$\begin{aligned} \frac{dR}{dt} = \frac{( \frac{P_0 R_0^3}{R^3} - P_e)R^4}{12 \eta R_0^2 {s_0}}. \end{aligned}$$.

### Model implementation

#### Solving the heat equation

The initial particle temperature was assumed to be uniform throughout the particle and equal to the temperature of the particle’s surrounding environment (the Unit 1 reactor). By imposing this initial condition and the aforementioned boundary conditions, Eq. (1) was solved using a dimensionless analytical solution for a convectively cooled sphere:

14 $$\begin{aligned} \theta ^* = \sum _{n=1}^{\infty } C_n exp(- \zeta _n^2 Fo) \frac{1}{\zeta _n r^*} sin(\zeta _n r^*), \end{aligned}$$

where $\theta ^*$ is dimensionless temperature, $C_n$ and $\zeta _n$ are constants whose derivations are given in Appendix B. The dimensionless variables of temperature, *T* , radial position, *r*, and time, *t*, were defined, respectively, as

15 $$\begin{aligned} \theta ^*= & {} \frac{T - T_\infty }{T_i - T_\infty }, \end{aligned}$$

16 $$\begin{aligned} r^*= & {} \frac{r}{r_p}, \end{aligned}$$

17 $$\begin{aligned} {\text {Fo}}= & {} \frac{k_b t}{ \rho _b c_b r_p^2}, \end{aligned}$$

where $T_i$ is the initial particle temperature and all other variables have been previously defined^41^.

#### Solving the bubble growth equation

The radial void growth, given in Eq. (13), was calculated using the numerical Euler method. Iterations were performed over a time step, *dt*, of $10^{-4}$ s with a spatial resolution of $10^{-8}$ m. Radial pore growth was terminated when the simulated viscosity of the melt surrounding the pore reached the glass transition temperature (the temperature at which the silicate melt changes to a polymer glass^42^), which was taken to be the temperature corresponding to a viscosity of 10^12^ Pa s^26^.

#### Simulating particle cooling

Initially, the one dimensional radial and temporal temperature and viscosity profiles of a molten sphere of composition similar to a ‘Type B’ CsMP were calculated, as outlined above. Subsequently, 10 bubble nucleation sites were randomly selected along a one-dimensional grid which had a length equal to the simulated particle radius. A bubble was then modelled to grow radially outwards from each selected site using Eq. (13). The viscosity values corresponding to the initial nucleation site of each bubble were used as the viscosity of $s_0$. This simulation was then repeated for numerous radial ‘probes’ into the simulated particle until the produced modelled bubble volume was equal to that observed in ‘Type B’ CsMP. This simulation was performed over a range of initial temperatures and particle radii, and the void diameter distributions for each set of initial conditions was obtained.

### Parameter selection

A complete list of the parameters used in our model is provided in Table 2. The selected parameters were chosen from the literature to closely match the properties of CsMP and their surrounding environment. The thermal conductivity, heat capacity and density of silicate ‘Type B’ particulate were taken to be the same as those of volcanic bombs with similar composition. It was assumed the particles were carried by the explosive shockwave, which was determined from CCTV footage of the blast, and thus had the same velocity^6^. The average elemental composition of the CsMP was obtained from recent studies^22,33,43^. These weight percentages, combined with the optimisation parameters in^38^, were then used to calculate the parameters *B* and *C* and calibrate the viscosity model. Since the particles were released into the surrounding environment after the hydrogen explosion, the external pressure experienced by the particle was taken to be atmospheric pressure. Considering the particles were in the reactor for approximately one day before the explosion, it was assumed they had reached thermal equilibrium with their surroundings. Therefore, the initial pressure of the bubbles within the CsMP was taken to be the estimated pressure of the PCV (the vessel where the CsMP formed) before the hydrogen explosion occurred. As bubbles close to the melt exterior experienced insignificant levels of growth in real ‘Type B’ CsMP, the initial radii of the modelled voids were randomly sampled from the observed distribution of circumferential void diameters. Finally, the external temperature and air velocity were obtained from weather reports of Fukushima on 12th March 2011, whilst the air viscosity and conductivity were assumed to be that of ambient air.

**Table 2:** Parameter values utilised in the presented model.

Description	Symbol	Value	Unit	Reference
		Particle parameters		
Thermal conductivity	$k_{p}$	1.5	$Wm^{-1} K^{-1}$	^26^
Heat capacity	$c_{p}$	1096	$J kg^{-1} K^{-1}$	^26^
Density	$\rho _{p}$	2400	$kgm^{-3}$	^26^
Particle velocity	$v_{p}$	360	$ms^{-1}$	^6^
		Viscosity Parameters		
Fit parameter	*A*	-4.55	–	^38^
Fit parameter	*B*	4583.1	*J*	–
Fit parameter	*C*	674.5	*K*	–
Glass transition temperature	$T_g$	951.4	*K*	–
		Bubble parameters		
External pressure	$P_e$	0.1 (1 atm)	*MPa*	^44^
Internal pressure	$P_0$	0.55	*MPa*	^5^
Initial void radius	$R_0$	random (<10)	$\mu m$	^21^
		Air parameters		
Prandtl number	Pr	0.71	–	^26^
External (air) temperature	$T_\infty$	280	*K*	^45^
Air viscosity	$\eta _g$	1.71 $\times 10^{-5}$	*Pa s*	^27^
Air velocity	$v_g$	1.6	$ms^{-1}$	^45^
Air conductivity	$k_g$	0.024	$Wm^{-1} K^{-1}$	^34^

## Results

### Void diameter distribution

**Figure 6:**
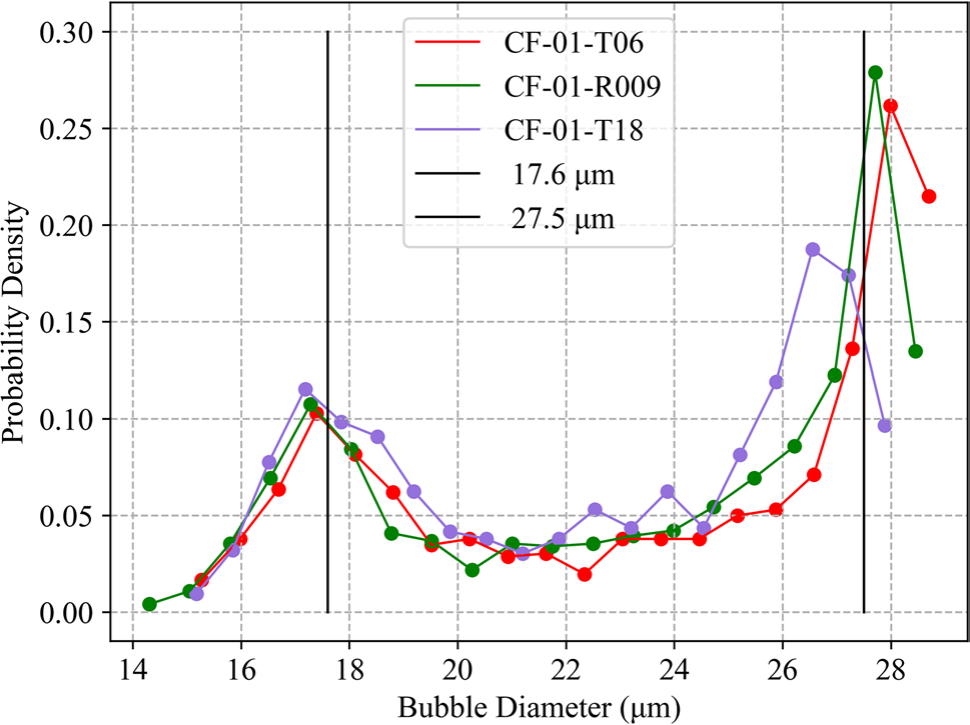
Apparent bi-modal void diameter distribution within the simulated particles comparable to CF-01-R009, CF-01-T18 and CF-01-T06 with respective diameters of 406.5 μm, 336.5 μm and 384.5 μm. The observed distribution was obtained using an initial pressure of 0.55 MPa and an initial temperature of 1900 K and shows peaks centred around 17.6 μm and 27.5 μm, respectively.

The simulated void diameter distribution for three simulated CsMP of different sizes is shown in Fig. 6. Like the observed void diameter distribution of physical ‘Type B’ CsMP, shown in Fig. 4, a bi-modal distribution is also produced by our model, indicating that the cooling profile was accurately captured. The first peak is centred, on average, at 17.6 μm, identical to the position of the first peak in Fig. 4. However, the second peak appears at a higher frequency and lower void diameter than the observed data. The likely reasons for this discrepancy are subsequently discussed.

### Temperature estimate

**Figure 7:**
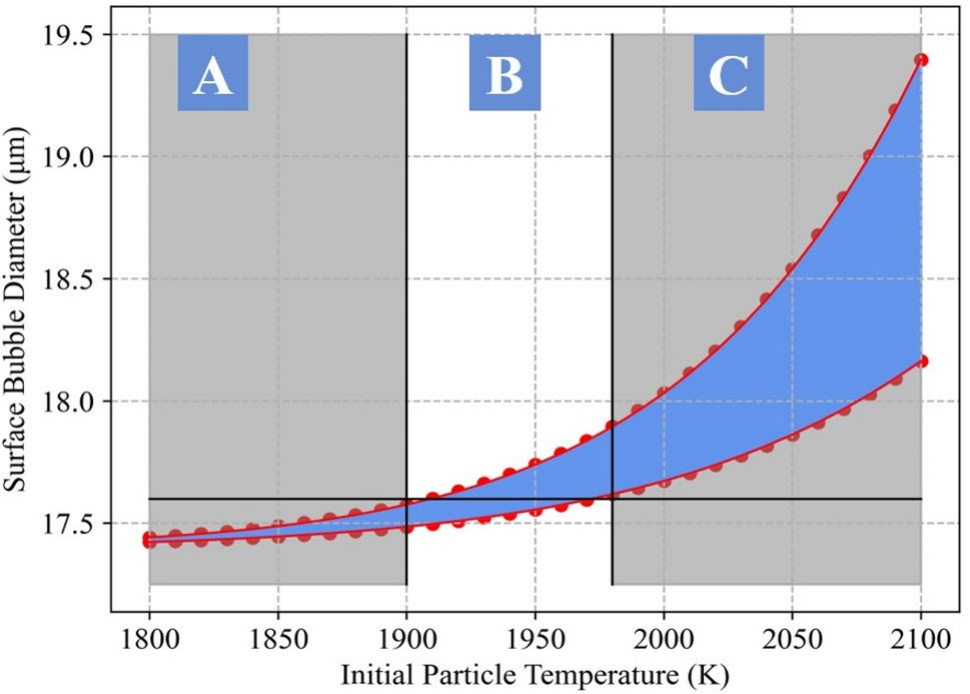
Plot of of surface bubble diameter against initial particle temperature used to estimate the temperature within reactor Unit 1. Region A marks the temperatures which were deemed too low, due to negligible central void growth, whereas region C marks those deemed too high, due to excess growth of surface level voids. Region B, however, marks the obtained estimated temperature range. The observed spread is due to the range of bubble diameters produced across the range of calibration parameters.

**Figure 8:**
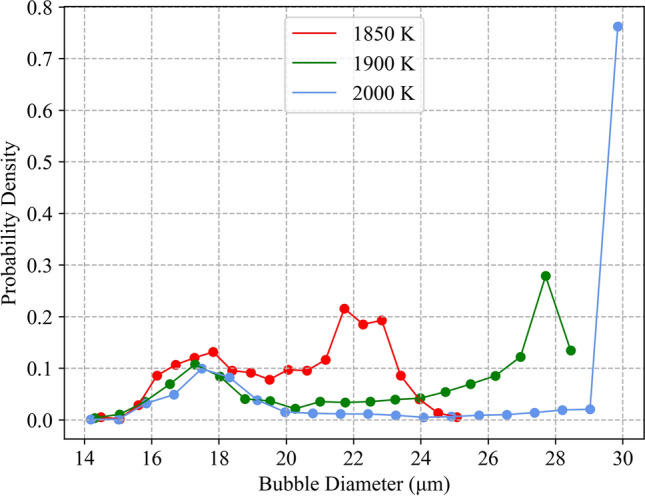
Plot of bubble diameter against probability density of the particle CF-01-R009, with radius 406.5 μm, at initial temperatures of 1850 K, 1900 K and 2000 K. The lower temperature corresponds to region A, where central bubbles experience minimal growth. The distribution at 1900 K displays the bi-modal distribution within the accepted model range for comparison. The highest temperature demonstrates the distribution above the predicted temperature range. In this case, most bubbles, including surface level bubbles, are able to reach a maximum size since the initial temperature is too high for quench-like cooling to occur.

Via investigation of void growth at various initial temperatures, the temperature of reactor Unit 1 was estimated to be between 1900 and 1980 K, marked by region B in Fig. 7. This estimate was established by varying the initial temperature of the modelled CsMP and determining the effects on void growth. With reference to Fig. 7, at temperatures above 1,900K (region C), the growth of surface level bubbles was significant. In reality, the surface level bubbles experienced negligible growth due to quench-like cooling. Hence, any temperature corresponding to a modelled situation where surface level voids grew was unrealistic, so was deemed an invalid estimate. The lower temperature bound was established to be the temperature at which negligible central bubble growth occurred, and the error in final bubble diameter was taken to be the range of bubble diameters obtained when using the maximum and minimum values of the calibration parameters. This is further demonstrated in Fig. 8, where it is evident that the characteristic bi-modal distribution breaks down outside of our estimated temperature range.

## Discussion

In order to minimise the complexity of the presented model, several assumptions were made. First, the assumption that CsMP were spherical and isotropic allowed for the reduction of a 3-dimensional problem into that of only 1 dimension. Many ‘Type B’ CsMP were well rounded by surface tension forces during transport and have aspect ratios close to 1^33^, thereby validating this approximation. However, irregularly-shaped ‘Type B’ particles have also been identified in the environment^8^, whose cooling was less accurately captured by our model. Due to their higher surface area, cooling of these more angular particles would have been faster^46^. As a result, they would be expected to have a higher number of surface level voids with smaller diameters due to the increased surface area and faster cooling.

**Figure 9:**
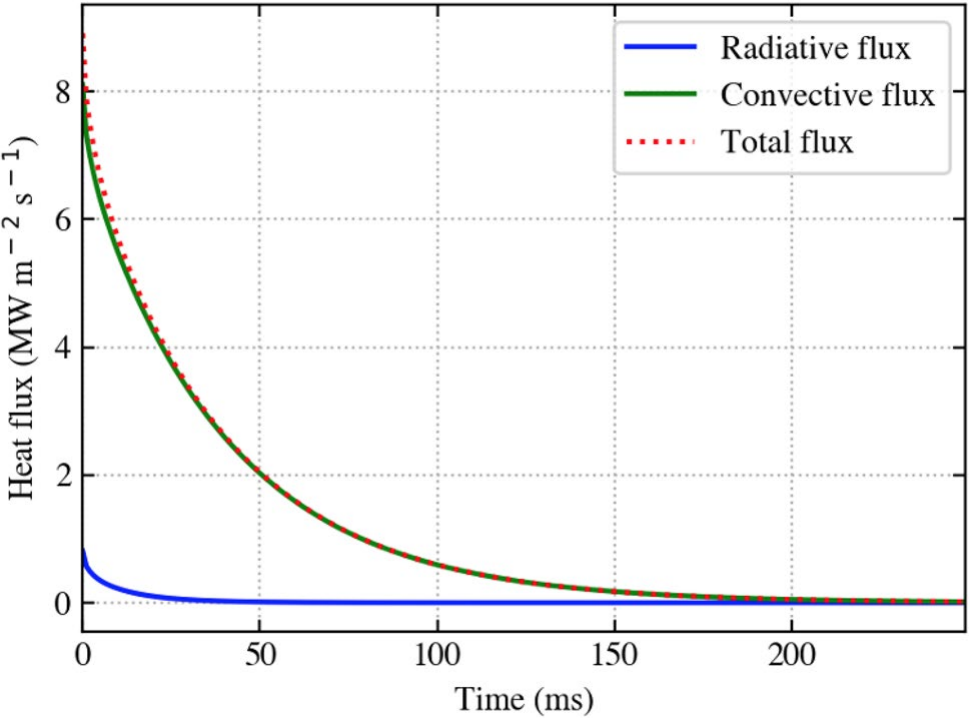
Convective and radiative heat flux calculated using the implicit solver py-pde^47^ for a 400 μm particle initially at 1960 K. Radioactive cooling is negligible except at the very start of cooling, where it is still dominated by the much greater convective flux.

Secondly, the modelled CsMP were assumed to cool via forced convection only. The py-pde python package^47^, which implicitly evaluates partial differential equations, was utilised to verify this assumption via calculation of surface heat flux due to both convective and radiative cooling. As seen in Figure 9, these calculations confirm the validity of this assumption, since radiative heat loss is comparatively very small initially and close to zero thereafter. In fact, the radiative cooling contribution was even smaller than that calculated, since the calculation assumed CsMP were emitting as perfect black bodies and consequently outputting the maximum radiative heat flux. This second assumption greatly reduced the computational complexity of the model, as it enabled the use of the faster analytical solution as opposed to the inefficient implicit solver.

It was also assumed that the melt viscosity was uniform across the bubble-melt interface. In reality, the radial temperature gradient resulted in a higher melt viscosity at the outermost edge of a bubble than at the innermost edge. This variation was found to be negligible on the scale of even the largest voids, and therefore allowed the viscosity value of the melt shell to be taken as the viscosity corresponding to the co-ordinate of the bubble centre.

In addition, the model did not account for the effects of crystal structure formation which, if present, acts to increase melt viscosity and restrict bubble growth. In volcanology, these effects are accounted for using the Einstein–Roscoe correlation^26^. However, for ‘Type B’ CsMP, the cooling timescale is much shorter than the crystallisation timescale^48^. Hence, while there may be a high level of nucleation within the ‘Type B’ CsMP, there is little-to-no crystal growth and the omission of such effects was accurate.

Furthermore, there was assumed to be a constant molar mass of gas inside growing bubbles. In fact, Martin et al.^21^, detected bright ‘halos’ of increased X-ray attenuation in EDS measurements caused by local differences in volatile (fission product) elements. These indicate that as bubbles grew during decompression, volatiles diffused in from the surrounding melt and were subsequently resorbed as the temperature dropped and the melt solubility increased causing Cs and Sr enrichment at the bubble margin. As a result, the number of moles of internal gas was not constant during bubble growth. This was not accounted for in this work, but future iterations of the model should take these processes into account in order to elucidate refined temperature regimes for ‘Type B’ CsMP formation. For example, the equations developed by Prousevitch et al.^39^, account for bubble growth due to a changing volatile concentration profile, which itself might be inferred from the concentrations of different volatile species in and around voids at different particle depths.

Finally, surface tension effects were neglected in our model due to their importance only in the very early stages of bubble growth, while their size is comparable to the nucleation size. This resulted in an idealised void-scale model that allowed us to directly investigate the effect of radial variations in melt viscosity on the internal texture of CsMP. During this period, bubbles grow slowly until passing some critical radius, after which time surface tension effects become negligible and the bubble can grow to its final size. Prousevitch et al.^39^, derived a formula for the critical radius, $R_{c r}$ by considering the internal bubble pressure and the saturation pressure of gas dissolved in the melt:

18 $$\begin{aligned} R_{c r}=\frac{2 \sigma }{\frac{c_o^2}{K_h}-p_m} \end{aligned}$$

where $\sigma$ is the surface tension of the melt, $c_o^2$ is the concentration of the dissolved gas, $K_h$ is Henry’s constant, and $p_m$ is the melt pressure. While we don’t consider volatile diffusion in our model, Eq. (18) provides an indication of the scales where the surface tension contribution is important. For the magmatic silicate melts that were introduced as analogues to molten CsMP ejecta, $R_{c r}=0.071 \upmu$ m^26^, which is significantly smaller than the bubble diameters calculated by our model. For central bubbles, with an origin attributed to the release of trapped gas from silicate fibres^21^, the assumption of negligible surface tension is valid since these had an initial radius much larger than $R_{c r}$. However, for surface level bubbles which grew initially from the amalgamation of gas molecules dissolved in the melt, this early period may still be significant. Future work is needed to extend the bubble growth model for ‘Type B’ CsMP to incorporate surface tension effects from the outset. We predict its inclusion would still preserve the characteristic bi-modal distribution, but might result in surface-level bubble diameters that are smaller than those calculated in this study.

**Figure 10:**
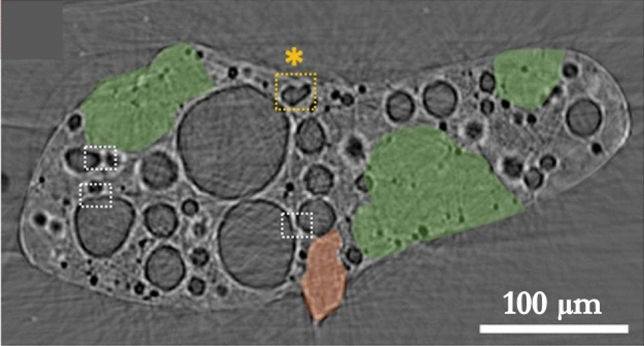
Synchrotron radiation XRT image of the inner structure of a ‘Type B’ CsMP. Marked by the white and yellow boxes are the locations where voids connect or coalesce, respectively. The Fe-rich region of the particle is highlighted in orange, while the green region marks Ca-rich, low porosity areas. From^21^.

As demonstrated in Fig. 6, the presented model successfully reproduced a bi-modal void diameter distribution comparable to that observed in real ‘Type B’ CsMP (Fig. 4). However, the simulation did not account for internal void coalescence, and as a result it was not possible to exactly match the relative frequency of the two peaks, nor their positions, to the real data. During the rapid depressurisation of a CsMP, internal bubbles grew until eventually adjacent bubbles coalesced and merged, resulting in central voids much larger than the maximum size calculated by our model. This process is visible in the interior of certain ‘Type B’ CsMP (Fig. 10) if the CsMP melt solidified before void coalescence was complete. These interactions between bubbles can be comfortably neglected when examining the formation of porous pyroclasts as the scale of maximally-grown bubbles is several orders of magnitude smaller than the clast size. This is not the case for the micron-scale ‘Type B’ CsMP. Thus, a complete analysis including coalescence is needed to accurately predict the final radii of the largest bubbles in ‘Type B’ CsMP. It is expected that this will increase the final radii of large voids whilst reducing their frequency, thereby recovering the void diameter distribution observed in real CsMP.

To summarise, whilst the presented study largely solves the problem by adapting and enhancing existing volcanic bomb cooling models to study ‘Type B’ CsMP formation, future work is needed to refine the model, particularly with regards to the proper treatment of surface tension and void coalescence in the bubble dynamics. For example, the equations presented by Prousevitch et al.^39^ account for surface tension as well as the changing volatile concentration profile. However these more complex calculations risk introducing numerical artefacts into the model which are difficult to distinguish from physical behaviour in nature^40^. Regardless, our approximate study clearly demonstrates the accuracy of the CsMP-volcanic bomb analogy, and confirms the hypothesis of Martin et al.^33^, who invoked the radial variation in cooling rate as the cause of a bi-modal void diameter distribution. Since the CsMP were molten in the instant preceding their environmental release, the temperature of reactor Unit 1 was expected to exceed the 1491 K melting point of the CsMP precursor material^49^ (Rockwool), as our estimate does. Our temperature estimate is also concordant with the lower bound imposed by the presence of Sb in ‘Type B’ CsMP, which implies the particles formed at temperatures greater than 1,860 K^16^, lending further credence to our results. Although temperatures in the reactor were known to be in excess of 3073 K during the core meltdown, the hydrogen explosion occurred $\sim$ 8 hours after this melting reportedly ended. In this time, venting of the suppression pool took place, which would have resulted in the inflow of air to the damaged RPV and PCV, causing the reactor temperature to fall to between those temperatures inferred by our model.

The analogy between ‘Type B’ CsMP and volcanic glassy airfall implies these reactor Unit 1 derived particulates may be brittle and easily friable, akin to pumice. However, the rapid quenching ($\sim$0.2 s) predicted by our model yields silicates with innate mechanical strength due to high internal residual stresses, very similar to Pele’s tears or Prince Rupert’s drops^23,50^. In addition, the tendency to minimize the total surface area of the gas-melt interface in the absence of shear (deformational) stresses results in the rounding of melt fragments, which further contributes to CsMP mechanical strength^33^. The characteristic timescale for this rounding process is given by;

19 $$\begin{aligned} \tau _{\text{ round } }=\frac{\eta r}{\Gamma }, \end{aligned}$$

where $\eta$ is melt viscosity, *r* melt radius, and $\Gamma$ surface tension (which is of order 10$^{-1}$ N m$^{-1}$). For a 200 μm particle cooling from 1900 K, $\tau _{\text {round}}\sim$ 0.5 ms. The time for an equally sized particle to cool to below the glass transition is $\sim$ 10 ms, which proves ‘Type B’ CsMP experienced significant rounding in the atmosphere before solidification. Their resistance to mechanical attrition invokes that they will be stable in the environment for a sustained period of time. However, the vitrified state of highly active Cs and UO$_2$ entrapped in the glassy matrix implies that the breakdown of ‘Type B’ CsMP represents a significant radiation hazard by exposing these elements and compounds^25^. Fortunately, the significant size and strength of the particulates mean they are unlikely to fragment further under surface environmental conditions; contrasted with the much smaller (1–10 μm) suite of radioactive ‘Type A’ material which is easily re-suspended and able to penetrate deep inside the lungs and possibly enter the bloodstream^51^.

TEPCO plans to commence decommissioning and dismantling operations at FDNPP Unit 1 in December 2021, a large part of which includes the mechanical milling and removal (via a robotic arm) of the fuel debris^33^. The Corium ‘slump’ of fuel and reactor core components melted through both the RPV and the PCV by the time the hydrogen explosion on 12th March, resulting in violent molten core-concrete interactions (MCCI). Comparison of the aforementioned magmatic systems associated with the nuclear meltdown implies that this debris is also likely to be extremely resistant to mechanical breakdown. The proposed removal methods are therefore expected to be significantly more challenging than in other reactor buildings, however, we foresee the risk of radioactive dust generation to be minimal due to mechanical properties of ‘Type B’ material outlined above.

Following the work of Benage et al.^26^, an improvement of our model upon earlier pyroclast cooling models is the inclusion of the relative velocity between the ejecta material and ambient air in the calculation of the Reynolds number. While out of the scope of this study, an investigation into the effect of the ambient air temperature and velocity on the final CsMP texture would provide useful information about the buoyant air entrainment of particles after the hydrogen explosion. This data could then be used to refine existing fallout dispersion models of the accident, such as the work by Yoshida et al.^20^, and identify possible areas of contamination.

The rounding of large ‘Type B’ CsMP after ejection lends credence to the spherical approximation used in this work. However, a significant number of Unit 1 particulates with irregular shapes have also been found in the environment^8^. In order to more accurately study the formation of this material, it is recommended to couple a finite element heat transfer simulation with the bubble growth model presented herein^39,40^. In addition, 3D XRT data of ‘Type B’ CsMP could be used to generate the mesh of the proposed simulation, with the actual coordinates of void nuclei being used for the lower-scale bubble growth model rather than the random sampling method utilised in this work.

As is the case with all mathematical modelling of physical systems, experimental validation is still required to confirm the findings of this study. We propose an experiment similar to those conducted by Okumura et al.^52^, and Kogure et al.^53^, where synthesis of smaller ‘Type A’ material was attempted in order to elucidate formation mechanisms for the Unit 2 derived particulates. Such a study might take the following form:

1. Suspend samples of precursor Rockwool insulation upon a heat resistant pedestal (e.g. ceramic) inside a pressurized, non-reactive cell (stainless steel or zirconium).
2. Connect the cell to a gas rig to control the pressure and the inflow of gases, including tracer gases such as hydrogen or deuterium.
3. Using an internal filament, melt the sample at the temperatures identified in this study.
4. Rapidly cool and depressurise the system using a turbo-pump, thereby quenching the melt.
5. Analyse the resulting material using focused ion beam (FIB), SEM, XRT, and X-ray fluorescence (XRF) techniques and compare the results to real ‘Type B’ CsMP samples.

## Supplementary Information

Supplementary Information.

## Data Availability

The datasets produced in this study are available in the GitHub repository: https://github.com/lior-carno/type-b-ejecta-model.git.
