# Supplementary material for: Modelling ‘Type B’ ejecta formation reveals reactor Unit 1 conditions during the Fukushima Daiichi Nuclear Disaster
Source: Sci Rep. 2023 Mar 6;13:3686. doi: 10.1038/s41598-023-30903-6 (PMC9988873; doi:10.1038/s41598-023-30903-6)
Supplement: Supplementary file 1 — Supplementary Information. [file 41598_2023_30903_MOESM1_ESM.pdf]

Modelling ‘Type B’ Ejecta Formation Reveals Reactor Unit 1 Conditions during the Fukushima Daiichi Nuclear Disaster

Lior A. S. Carno[1,*,+], Jack J. Turner[1,*,+], and Peter G. Martin[2,*]

[1]University of Bristol, School of Physics, HH Wills Physics Laboratory, Tyndall Avenue, Bristol, BS8 1TL, UK
[2]University of Bristol, Interface Analysis Centre, School of Physics, HH Wills Physics Laboratory, Tyndall Avenue, Bristol, BS8 1TL, UK
[*]lc17812@alumni.bristol.ac.uk, jt17874@alumni.bristol.ac.uk, peter.martin@bristol.ac.uk
[+]these authors contributed equally to this work

Appendices

A Derivation of the thin shell model

The following analysis of an overpressured viscous shell of thickness s was first presented by Sparks *et al.*[1]. Using spherical coordinates, assume the Navier-Stokes equations

$$\text{continuity:} \quad \frac{1}{r}\frac{\partial}{\partial r}\left(r^2 u\right) = 0 \tag{1}$$

$$\text{r-momentum:} \quad \frac{\partial u}{\partial t} + u\frac{\partial u}{\partial r} = -\frac{1}{\sigma}\frac{\mathrm{d}P}{\mathrm{d}r} + \nu\frac{1}{r^2}\frac{\mathrm{d}}{\mathrm{d}r}r^2, \tag{2}$$

$$\frac{\mathrm{d}u}{\mathrm{d}r} - \frac{2u}{r^2} \tag{3}$$

$$\theta\text{-momentum:} \quad 0 = -\frac{1}{\sigma r}\frac{\mathrm{d}P}{\mathrm{d}\theta}, \tag{4}$$

$$\lambda\text{-momentum:} \quad 0 = \frac{1}{\rho r \sin\theta}\frac{\mathrm{d}P}{\mathrm{d}\theta} \tag{5}$$

where ∂ are partial derivatives. This assumes there is radial symmetry. The bubble is tiny enough and the radial velocities so small that the conditions at infinity can be neglected, and so the bubble only interacts with the local pressure field.

From the continuity equation 1 it follows that r^2u must be a function of t (since $\partial/\partial r = 0$ and must therefore be a constant)

$$r^2 u = f(t) \Rightarrow u = f(t)/r^2. \tag{6}$$

The particle velocity at the bubble interface $r = R(t)$ is $\frac{d}{dt}R(t) = R'(t)$. Therefore

$$u = R' = \frac{f(t)}{R^2} \Rightarrow u = \frac{R^2 R'}{r^2}. \tag{7}$$

This form can be substituted into the r-momentum equation.

Similarly, the Navier-Stokes equations can be assumed to produce an equation for P:

$$P = \rho \left(\frac{2RR' + R^2 R''}{r^2} - \frac{R^4 R'^2}{r^5} \right) + P(\alpha, t). \tag{8}$$

Assuming that $P = P_i$ for $r < R$, the internal pressure, and $P = P_e$ for $r > R + s$, the external pressure, in this case two equations for continuity of stress are valid applied across each of the two interfaces:

$$-P_i = P|_{r=R} + 2\eta \left.\frac{\partial u}{\partial r}\right|_{r=R} \tag{9}$$

$$-P_e = P|_{r=R+s} + 2\eta \left.\frac{\partial u}{\partial r}\right|_{r=R+s} \tag{10}$$

where η is the melt viscosity. Conservation of mass allows that the mass of the shell may be expressed as the difference between the mass of the whole and the mass of the bubble:

$$(R+s)^3 - R^3 = \frac{3M}{4\pi\rho} \tag{11}$$

and the ideal gas law also applies. Substituting 8 into the equations for internal and external pressure the following expression can be obtained:

$$P_i - P_e = r \left(\frac{3R'^2}{2} - R'^2 \left(\frac{2R}{R+s} - \frac{R^4}{2(R+s)^4} \right) + R'' \left(R - \frac{R^2}{R+s} \right) \right) + 4\eta R' \left(\frac{1}{R} - \frac{R^2}{(R+s)^3} \right). \tag{12}$$

Expanding using the binomial theorem yields the approximation

$$\frac{P_i - P_e}{\rho} \approx \frac{3s^2 R'^2}{R^2} + sR'' + 12 \frac{\eta s R'}{R^2}. \tag{13}$$

Binomial expansion of 11 will result in an approximation

$$s \sim \frac{M}{4\pi\rho R^2}. \tag{14}$$

Using this expression follows

$$\frac{P_i - P_e}{\rho} \approx \frac{3M^2}{(4\pi\rho)^2} \frac{R'^2}{R^6} + \left(\frac{M}{4\pi\rho} \right) \frac{R''}{R^2} + \frac{3M\eta}{\pi\rho} \frac{R'}{R^4}. \tag{15}$$

Neglecting those terms related to inertial stress in the viscous analysis and using the binomial expansion 11, equation 15 can be re-expressed as

$$P_i - P_e = \frac{12\eta s R'}{R^2}. \tag{16}$$

B Analytical Solution Constants

From Recktenwald *et al.*[2], The coefficient ζ_n is obtained by calculating the roots of the equation

$$1 - \zeta_n \cot(\zeta_n) = Bi \tag{17}$$

where Bi, is the biot number, taken to be

$$Bi = \frac{hR_p}{k_p} \tag{18}$$

where h is the heat transfer coefficient, R_p is the particle radius and k_p is the thermal conductivity. These constants were subsequently used to calculate C with

$$C_n = \frac{4\left[\sin(\zeta_n) - \zeta_n \cos(\zeta_n)\right]}{2\zeta_n - \sin(2\zeta_n).} \tag{19}$$

References

1. Barclay, J., Riley, D. S. & Sparks, R. S. J., Analytical models for pore growth during decompression of high viscosity magmas, *Bulletin of Volcanology* **57**, 422–431 (1995).

2. Recktenwald, G., Transient, One-Dimensional Heat Conduction in a Convectively Cooled Sphere. (Portland State University, Oregon, 2006).
